# Type A Quadricuspid Aortic Valve Infective Endocarditis Complicated by Multiple Aortocardiac Fistulae: Case Report and Brief Literature Review

**DOI:** 10.1155/2017/2865305

**Published:** 2017-05-18

**Authors:** Amine Ghalem, Mohammed Bachrif, Anass Hbali, Mostapha Beghi, Nabila Ismaili, Noha El Ouafi

**Affiliations:** ^1^Mohammed VI University Hospital, Oujda University, BP 4806, 60049 Oujda, Morocco; ^2^Department of Cardiology, Mohammed VI University Hospital, 60049 Oujda, Morocco

## Abstract

Aortocardiac fistulae (ACF) are exceptionally due to infective endocarditis; they are usually congenital, posttraumatic, or complicate aortic dissection. In infective endocarditis setting, their presence should prompt urgent surgery as patients can deteriorate rapidly. We report the case of a 78-year-old female patient with the first ever reported quadricuspid aortic valve infective endocarditis complicated by multiple aortocardiac fistulae. Additionally, we provide a brief review of ACF, in infective endocarditis and quadricuspid aortic valve.

## 1. Introduction

Aortocardiac fistulae are exceptionally due to infective endocarditis; they are usually congenital, posttraumatic, or complicate aortic dissection [1, 2]. In infective endocarditis setting, the rare presence of aortocardiac fistulae mandates urgent surgery given their poor prognosis [3].

Our case describes a 78-year-old female patient with the first ever reported, to the best of our knowledge, quadricuspid aortic valve infective endocarditis complicated by multiple aortocardiac fistulae.

## 2. Observation

**Figure 1:**
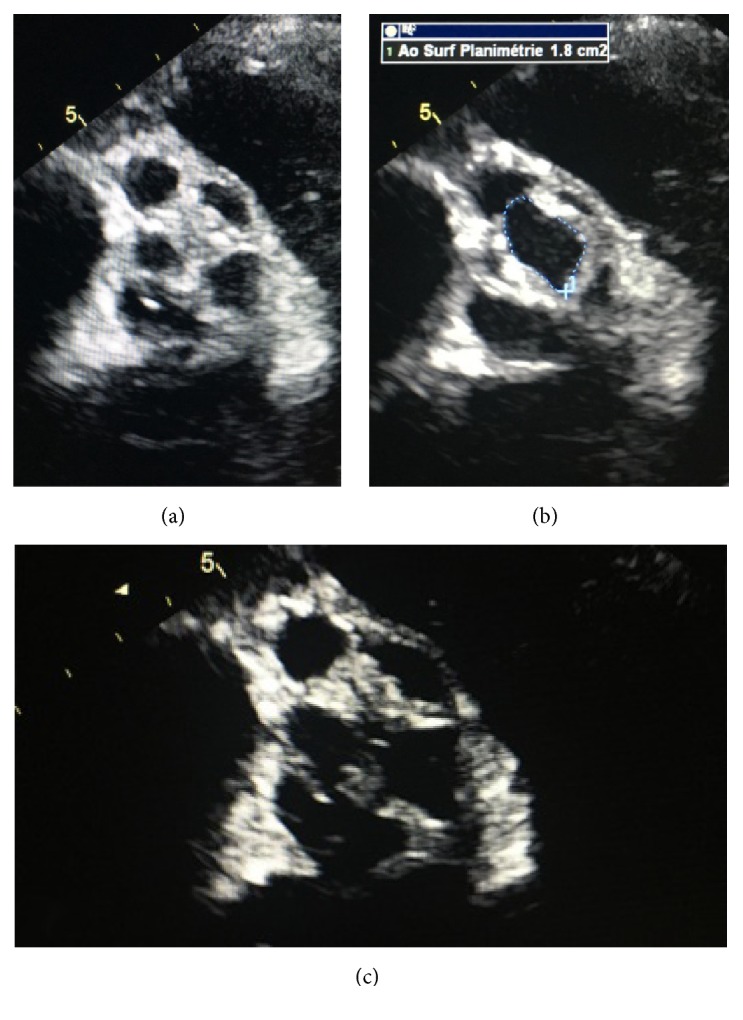
TTE: parasternal short axis view showing type A quadricuspid aortic valve in systole (a) and diastole with a planimetric aortic valve area of 1.8 cm^2^ (b). (c) shows type A quadricuspid aortic valve during systole along with two fistulae, the first between the left sinus of Valsalva and the left atrium (LA) and the second between an aortic periannular abscess and LA.

**Figure 2:**
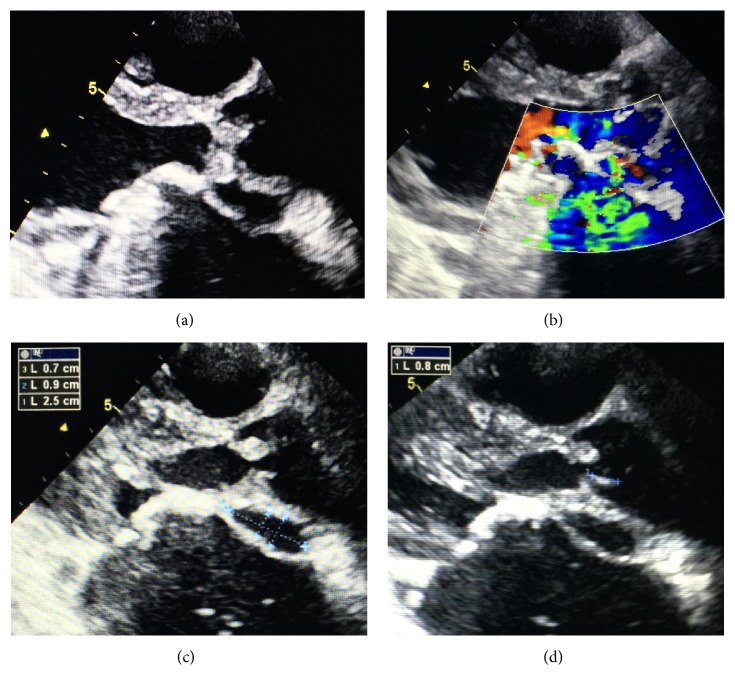
TTE: parasternal long axis view showing aortic periannular abscess communicating with the left atrium without (a, c) and with color Doppler imaging (b) and aortic vegetation (d).

A 78-year-old female with a history of severe mitral stenosis and mild aortic stenosis with subsequent mechanical mitral valve replacement in 2001 and a cholecystectomy in 2015 presented to our hospital with prolonged fever, exertional dyspnea, and orthopnea. Upon presentation, vitals were as follows: oral temperature of 38.3°C, blood pressure of 120/78 mmHg, heart rate of 105 beats/min, and respiratory rate of 26 breaths/min, with an oxygen saturation of 92% on room air. The patient was alert and oriented. Physical examination revealed signs of acute decompensated heart failure and a grade 4/6 continuous murmur in the aortic focus with normal prosthetic heart valve sounds. ECG showed atrial fibrillation. Laboratory tests were remarkable for elevated levels of NT-proBNP, C-reactive protein, erythrocyte sedimentation rate (ESR), and white blood cell count (WBC). Chest X-ray showed signs of pulmonary edema. Transthoracic echocardiography (TTE) revealed normal-functioning mitral prosthetic valve, type A quadricuspid aortic valve (QAV), aortic valve vegetation with aortic periannular abscess, severe aortic regurgitation (AR), and mild aortic stenosis. Furthermore, we evidenced two existing interruptions of tissue continuity between the aortic root and left atrium (LA) traversed by a turbulent flow inside revealing abscess and left sinus of Valsalva fistulization to LA (Figures 1 and 2).

**Figure 3:**
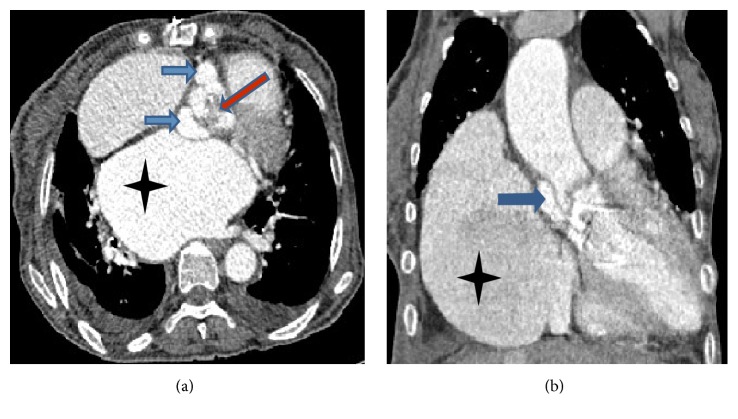
CT displays (a) on axial plane an irregular, deformed aortic valve, in which there is hypodense material (red arrow), two pseudoaneurysms (blue arrows), and dilated left atrium (black star). (b) Coronal reconstruction showing a pseudoaneurysm at the base of the ascending aorta (blue arrow) and dilated left atrium (black star).

The patient declined to perform transesophageal echocardiography (TEE). Serial blood cultures resulted positive for* Enterococcus faecalis*. Aortic valve infective endocarditis (IE) was established. The patient was started on amoxicillin, gentamicin, and intravenous furosemide. Multislice computed tomography (MSCT) displayed two pseudoaneurysms and hypodense material, suggestive of an abscess, in the aortic root (Figure 3).

Cerebral computed tomography scan (CT) was normal. Abdominal CT scan delineated splenic infarct. Given the bacteria incriminated in this case of IE, we performed a colonoscopy that was unremarkable. The hospital course was favorable with the disappearance of heart failure signs and apyrexia after the first week of treatment, along with regression of biological inflammatory syndrome. At this stage, we discussed cardiac surgery with the patient and her family who preferred conservative medical management. Unfortunately, on the 28th day after admission, the patient presented brutal, severe hypotension, and severe bradycardia followed rapidly by cardiac arrest. The patient died despite our best cardiopulmonary resuscitative efforts.

## 3. Discussion

The etiology of aortocavitary fistulas, abnormal connections between the aorta and cardiac cavities, is diverse. They can be congenital, caused by dissecting aneurysms and aortic dissection, secondary to blunt chest trauma, and they can also be seen after cardiac surgery especially operations involving the aorta or the aortic valve. Another cause, which is exceptional, is infective endocarditis (IE) [1, 2]. In a multicenter study of 4681 episode of IE, aortocardiac fistulae were diagnosed only in 1.6% of cases [4]. From a pathophysiological perspective, aortocardiac fistulae caused by IE are due to the extension of the infection to the perivalvular structures (aortic annulus and/or the mitral-aortic intervalvular fibrosa) with the subsequent creation of an abscess or a pseudoaneurysm that ruptures into cardiac chambers [4, 5]. The four cardiac chambers and the three sinuses of Valsalva are equally involved in the fistulous tract [4]. Our patient had quadricuspid aortic valve (QAV) IE complicated by two aortocardiac fistulae involving the left atrium. QAV is a rare abnormal development of semilunar valves that has been reported in 0.008%, 0.043%, and 1% in, respectively, autopsy series, two-dimensional TTE, and in aortic valve surgery [6]. However, thanks to advances in cardiac imaging technics, more cases have been reported. It is more often than not a solitary abnormality but is associated with patent ductus arteriosus, ventricular septal defect, pulmonary stenosis, hypertrophic cardiomyopathies, subvalvular and supravalvular aortic stenosis with left coronary atresia, and coronary ostia obstruction in nearly 10% of the cases [7]. AR is the most frequent complication of QAV accounting for 75% of the cases, where 9% have both aortic stenosis and regurgitation (isolated aortic stenosis is very rare) and 16% have normal-functioning QAVs [6, 8].

Echocardiography, especially TEE, is the preferred imaging technique for the detection of aortocardiac fistulae and QAV [4, 7]. Echocardiographic high diagnostic performance for aortocardiac fistulae is explained by the significant pressure gradient between the aorta and the cardiac chamber, which results in a highly turbulent flow across the fistulous formation readily detectable by continuous and color Doppler mapping even in the case of small fistulas [4]. In QAV, echocardiography shows an X-shaped pattern during diastole and a rhomboidal image in systole [7] (Figure 1). It is worth noting that in aortic IE, MSCT is an excellent imaging technique as it not only allows detection of abscesses/pseudoaneurysms—like in our case—with a similar diagnostic accuracy to TEE but also provides probably more information regarding the extent and consequences of any perivalvular extension, including the anatomy of pseudoaneurysms, abscesses, and fistulas. Additionally, in aortic IE, CT may help surgical planning [3].

Aortocardiac fistulae bear a poor prognosis; in the study previously mentioned, over 60% of patients with aortocardiac fistulae developed significant heart failure and over 40% died [4]. Thus, urgent surgery, along with antibiotics, is the treatment of choice in most cases; although some institutions send patients to surgery after completion of antibiotherapy unless other complications arose [2, 3, 5]. The primary objectives of surgery are total removal of infected tissues, replacement of the aortic valve using a mechanical or biological prosthesis, and closing of the fistula. Regarding the choice of the prosthesis to use, expert opinion favors cryopreserved or sterilized homografts [3]. In our case, the patient and her family refused surgery.

Only a few cases of EI involving QAV have been reported [9]. However, to the best of our knowledge, our case is the first to describe an association between QAV infective endocarditis and multiple aortocardiac fistulae. Also, two other findings were peculiar in our case; the first is the fact that the patient had a history of aortic stenosis without aortic regurgitation, which is very rare, the massive AR discovered by echocardiography being most likely secondary to IE. The second is the fact that our patient had type A QAV (all four cusps being of equal size) according to Hurwitz and Roberts classification, which is the variant less prone to IE because of the lack of asymmetry or flow disturbance [6]. Previously to our case, only Matsukawa and al. presented a bacterial endocarditis in a 75-year-old patient diagnosed with type A QAV [10].

## 4. Conclusion

Few QAV infective endocarditis cases have been reported. Our report is the first one to describe a unique association between QAV IE and multiple aortocardiac fistulae, which are dangerous complications bearing frightening prognosis. Echocardiography, especially TEE, is usually the preferred imaging technic for both QAV and aortocardiac fistulae, especially in African countries where MSCT is not always available. In the setting of IE, the presence of aortocardiac fistulae prompts urgent surgical intervention as patients can deteriorate rapidly.

## References

[1] John E. S., Boyer J., Ledzian B., Steward H., Moro R., Bittner H. B. (2014). A rare case of sinus of valsalva-right atrial fistula secondary to an abscess perforation from underlying aortic valve endocarditis. *Journal of Cardiothoracic Surgery*.

[2] Boutayeb A., Rhissassi J., Saghi G., Benyoussef H., Maazouzi W. (2011). Les fistules aorto-atriales droites sur double localisation endocarditique aortique et tricuspide À propos dun cas avec revue de la littérature. *Chirurgie Thoracique Cardio-Vasculaire*.

[3] Habib G., Patrizio L., Antunes M. J. (2015). 2015 ESC guidelines for the management of infective endocarditis: the task force for the management of infective endocarditis of the european society of cardiology (ESC). *European Heart Journal*.

[4] Anguera I., Miro J. M., Vilacosta I. (2005). Aorto-cavitary fistulous tract formation in infective endocarditis: clinical and echocardiographic features of 76 cases and risk factors for mortality. *European Heart Journal*.

[5] Ouafi A., Zeriouhi F., Chikhi F. (2011). Aortic-left atrium fistula: a rare and dangerous complication of the infective endocarditis. *Moroccan journal of Cardiology*.

[6] Malviya A., Jha P. K., Ashwin, Mishra J., Srivastava P., Mishra A. (2016). Quadricuspid aortic valve—a case report and literature review. *Egyptian Heart Journal*.

[7] Aboitiz-Rivera C. M., Blachman-Braun R., Lanza M. F. (2016). Quadricuspid aortic valve: an unexpected echocardiographic finding. *Medical Ultrasonography*.

[8] Yuan S.-M., Yan S.-L. (2016). Quadricuspid aortic valve: a case report. *Cor et Vasa*.

[9] Dagnegård H. H., Røpcke D. M., Lund J. T. (2016). Endocarditis in a quadricuspid aortic valve. *The Annals of Thoracic Surgery*.

[10] Matsukawa T., Yoshii S., Hashimoto R., Muto S., Suzuki S., Ueno A. (1988). Quadricuspid aortic valve perforation resulting from bacterial endocarditis–2-d echo-and angiographic diagnosis and its surgical treatment. *Japanese Circulation Journal*.

